# A Rare Case of Pseudohypercalcemia Associated with Multiple Myeloma

**DOI:** 10.24546/0100495981

**Published:** 2025-05-30

**Authors:** CHIHARU MISHIMA, KIMIKAZU YAKUSHIJIN, HIDENORI FUKUOKA, RURI TAKAHASHI, YURI OKAZOE, MIKI JOYCE, SAKUYA MATSUMOTO, RINA SAKAI, YUMIKO INUI, KEIJI KURATA, HIRONOBU MINAMI

**Affiliations:** 1 Division of Medical Oncology/Hematology, Department of Medicine, Kobe University Hospital, Kobe, Japan; 2 Division of Diabetes and Endocrinology, Department of Medicine, Kobe University Hospital, Kobe, Japan

**Keywords:** Pseudohypercalcemia, Multiple myeloma, Immunoglobulins, Ionized calcium

## Abstract

We report a rare case of pseudohypercalcemia associated with multiple myeloma in a 77-year-old woman. Despite elevated albumin-corrected calcium levels (12.6 mg/dL), ionized calcium levels remained normal (1.25 mmol/L). Differential diagnoses excluded common causes of hypercalcemia, and the findings suggested calcium binding to negatively charged immunoglobulins and confirmed pseudohypercalcemia due to IgG-type myeloma. Treatment with isatuximab plus dexamethasone normalized albumin-corrected calcium levels as IgG levels decreased. This report highlights the importance of recognizing pseudohypercalcemia to prevent misdiagnosis of true hypercalcemia due to myeloma. Measuring ionized calcium levels is crucial for accurate diagnosis when hypercalcemia is suspected without corresponding clinical symptoms.

## INTRODUCTION

Hypercalcemia is a biochemical abnormality that is frequently encountered in daily clinical practice and has various potential causes. Primary hyperparathyroidism and malignancies are known to be the major causes of hypercalcemia (1, 2), and hypercalcemia has various clinical manifestations. Mild hypercalcemia is often asymptomatic and does not cause complications; however, severe hypercalcemia (>14 mg/dL) can lead to acute renal failure, mental impairment, convulsions, and arrhythmias, requiring prompt treatment (3).

Multiple myeloma (MM) is a hematological malignancy characterized by the proliferation of abnormal plasma cells. In patients with multiple myeloma, an imbalance between the functions of osteoclasts and osteoblasts in the myeloma bone marrow leads to hypercalcemia. Approximately 30% of patients diagnosed of multiple myeloma present with hypercalcemia (4). As hypercalcemia is a multiple myeloma defining event (MDE), it serves as an important diagnostic indicator for multiple myeloma, as it reflects the presence of plasma cell-related bone disease (5). Although hypercalcemia is a well-known feature of multiple myeloma, pseudohypercalcemia is a rare phenomenon that can complicate the diagnostic process (6, 7). Pseudohypercalcemia is defined as an elevation in total calcium levels, while ionized calcium remains within the normal range. Since ionized calcium is the physiologically active form, pseudohypercalcemia does not lead to clinical symptoms typically associated with hypercalcemia (8). Here, we report a rare case of pseudohypercalcemia in a patient with symptomatic myeloma.

## CLINICAL CASE

A 77-year-old woman had been monitored for IgG-λ monoclonal gammopathy of undetermined significance (MGUS) at a nearby hospital. Routine laboratory tests revealed the progression of anemia and elevated serum total calcium and immunoglobulin IgG levels. She was referred to our hospital with a suspected progression to symptomatic myeloma. She was alert and oriented, with no other significant physical findings except for a Levine II/VI systolic heart murmur at the right upper sternal border. She had a history of chronic hepatitis C that was resolved with interferon therapy and no history of fractures. Other clinical symptoms such as gastrointestinal, renal, musculoskeletal, and cardiovascular manifestations were absent. Physical examinations revealed no apparent neurological abnormalities. Laboratory tests revealed a low serum albumin (3.6 g/dL) and a high β_2_-microglobulin concentration (3.1 mg/dL). The patient was diagnosed with stage I myeloma according to the International Staging System (ISS). Tests also revealed a low serum hemoglobin concentration (9.8 g/dL) and a significant increase in IgG levels (5,208 mg/dL). Her serum kappa/lambda free light chain (FLC) ratio decreased (FLC κ <0.50 mg/L, FLC λ 81.80 mg/L, and κ/λ ratio <0.006), and her serum total calcium concentration was 12.2 mg/dL and albumin-corrected calcium concentration was 12.6 mg/dL.

Whole-body computed tomography (CT) revealed an osteolytic lesion in the right ischium, and bone marrow aspiration and biopsy revealed that the proportion of abnormal plasma cells was 7%, which was less than a criterion for the diagnosis of myeloma (10%). However, considering the increase in concentration of non-IgM M-protein (≥3000 mg/dL), osteolytic lesions and anemia as MDE, she was diagnosed with symptomatic myeloma. In the absence of hypercalcemia symptoms, such as polyuria or abnormal electrocardiograms, further evaluation of hypercalcemia was conducted. Laboratory tests revealed a normal ionized calcium concentration (1.25 mmol/L), in contrast to a high albumin-corrected calcium concentration. Intact parathyroid hormone (PTH, 41 pg/mL) and 1, 25-dehydroxy vitamin D3 level (38 pg/mL) levels were also within normal ranges, and tumor markers such as parathyroid hormone related peptide (PTHrP), squamous cell carcinoma-related antigen (SCC), and cytokeratin 19 fragment (CYFRA 21-1 antigen) were negative. Differential diagnoses such as hyperparathyroidism, malignancy, and vitamin D toxicity were excluded, which lead to a diagnosis of pseudohypercalcemia associated with myeloma. We monitored her pseudohypercalcemia by measuring the ionized calcium concentration due to high albumin-corrected calcium concentration. The patient initially opted for low-intensity therapy at a local hospital and was then transferred.

**Figure 1:**
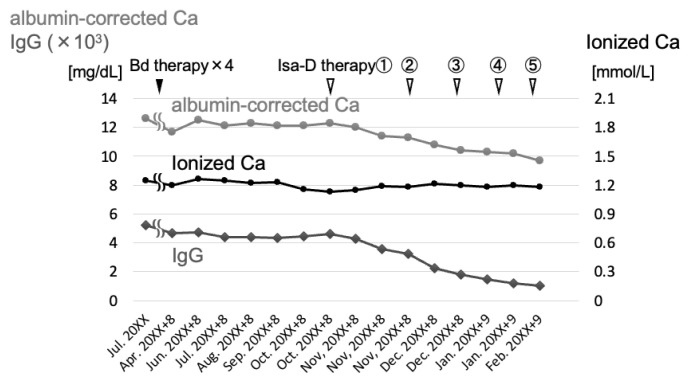
Changes in serum calcium and IgG levels after transfer to our hospital Switching from ineffective bortezomib and dexamethasone (Bd) therapy to isatuximab plus dexamethasone (Isa-D) therapy resulted in a successful reduction in IgG levels, accompanied by a corresponding decrease in albumin-corrected calcium concentrations. Ionized calcium levels remained stable within the normal range and showed no significant changes.

Seven years later, the patient returned to our hospital for further treatment. Informed consent was obtained from the patient for publication of this case report. Increased levels of IgG and pseudohypercalcemia as shown in Figure 1. Bortezomib plus dexamethasone was initiated but was ineffective. We then switched to isatuximab plus dexamethasone, which resulted in a decrease in IgG levels and a corresponding, parallel decrease in the albumin-corrected calcium concentration, leading to its normalization. The ionized calcium concentration remained stable and within the normal range (Figure 1). Notably, the discrepancy between albumin-corrected and ionized calcium concentrations was resolved.

## DISCUSSION

In myelomas, neoplastic plasma cells in the bone marrow produce monoclonal proteins that are sometimes associated with hyperviscosity syndrome, which leads to organ damage and various symptoms. MDEs include hypercalcemia, renal failure, anemia, and bone lesions “CRAB features” and are important for diagnosing symptomatic myeloma (5); therefore, hypercalcemia should be carefully monitored.

Hypercalcemia typically causes clinical manifestations such as polyuria, polydipsia, dyspepsia, fatigue, depression, muscle weakness, and constipation, which require appropriate treatment (3). In severe cases (>14 mg/dL), hypercalcemia can lead to dehydration, abdominal pain, vomiting, acute renal failure, impaired consciousness, and seizures (3). Despite the elevated serum total calcium and albumin-corrected calcium levels in our patient, no symptoms of hypercalcemia were observed. In this case, a certain portion of serum calcium was likely bound to the abnormally high levels of IgG, which may have contributed to a reduction in the proportion of ionized calcium. This suggests that IgG binding to calcium could have played a role in preventing an increase in ionized calcium levels, thereby potentially mitigating symptoms typically associated with hypercalcemia. The severity of symptoms generally correlates with calcium levels and the rate of increase in serum calcium levels. In patients with chronic hypercalcemia, symptoms, including neurological manifestations, may remain mild (2). Even if a patient presents with true hypercalcemia, the absence of symptoms could still be explained by its moderate severity and gradual progression. In general, hypercalcemia is classified into two categories, PTH-mediated and non-PTH-mediated, depending on serum PTH levels (3). PTH-mediated hypercalcemia, which is characterized by elevated PTH levels, is typically caused by primary hyperparathyroidism. In our patient, the PTH and ionized calcium levels were within the normal range, which excluded hyperparathyroidism. Non-PTH-mediated hypercalcemia is most often associated with malignancies including multiple myeloma, although other causes, such as sarcoidosis, tuberculosis, endocrine disorders (e.g., adrenal insufficiency and pheochromocytoma), drug-induced hypercalcemia, vitamin D supplementation, and familial hypercalcemia, should also be considered (3). After excluding these conditions, pseudohypercalcemia was suspected in our patient, as multiple myeloma remained the only plausible cause.

**Figure 2:**
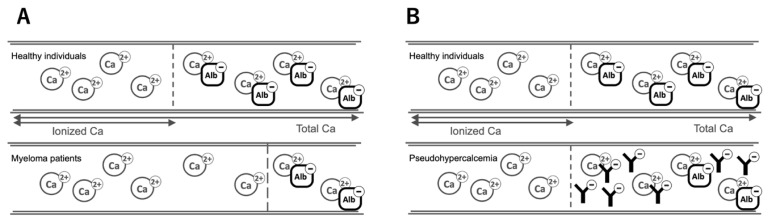
Distribution of total calcium: Ionized and albumin-bound fractions in serum (A) Approximately 45% of serum calcium is bound to negatively charged albumin, while the remaining half exists as ionized calcium, the physiologically active form. In patients with hypoalbuminemia, the amount of calcium bound to albumin decreases, which necessitates the use of albumin-corrected calcium concentrations. (B) In this patient, despite an elevated albumin-corrected calcium concentration, the ionized calcium level remained within the normal range, which suggested the binding of calcium to abnormally proliferated, negatively charged immunoglobulins.

Serum calcium concentrations are generally measured using routine blood tests. Approximately 45% of serum calcium is protein-bound (mainly albumin), 10% is bound to anions such as citrate and phosphate, and the remaining 45% is free or ionized calcium, which is physiologically active (1). Alterations in serum protein levels can affect total calcium concentration and lead to discrepancies in ionized calcium levels. Blood pH also influences the binding of calcium to albumin, and a lower pH reduces this binding. However, ionized calcium levels remain unaffected by changes in pH (1); thus, ionized calcium levels should be measured directly for accurate evaluation. In our case, serum total calcium and albumin-corrected calcium were elevated (12.2 mg/dL and 12.6 mg/dL, respectively); therefore, ionized calcium was originally thought to be increased as shown in Figure 2A. However, in contrast to the high albumin-corrected calcium concentration, the ionized calcium level remained normal (1.25 mmol/L), which confirmed pseudohypercalcemia with no pathological significance.

To date, there have been very few reports of pseudohypercalcemia associated with myeloma (6, 7, 9, 10). Pseudohypercalcemia in multiple myeloma is associated with abnormal immunoglobulins that bind to calcium. Among the limited reports, most cases of pseudohypercalcemia have been associated with IgG-type myeloma, followed by IgA-type myeloma (9). In this patient, as IgG levels decreased with effective treatment, the albumin-corrected calcium concentration also declined in parallel, while ionized calcium levels remained unchanged, which suggested that calcium bound to IgG (Figure 2B). However, the decrease in calcium levels may also have resulted from the successful treatment of multiple myeloma, leading to reduced bone resorption. As calcium homeostasis is tightly regulated through the intestines, kidneys, and bones (11), and since renal function was not impaired in this patient, the calcium released from IgG might have been processed through renal excretion or deposition into bone, contributing to the normalization of serum calcium levels after treatment.

In a rare case of myeloma associated with concurrent hyperviscosity syndrome and pseudohypercalcemia, the patient with elevated serum calcium levels and impaired consciousness underwent treatment for hypercalcemia, which proved to be ineffective. Subsequent plasma exchange for hyperviscosity syndrome resulted in rapid resolution of impaired consciousness (6). Another case involved a patient with myeloma with vitamin D deficiency who exhibited a secondary increase in PTH levels and was misdiagnosed with hyperparathyroidism. Despite the low ionized calcium levels, the patient’s plasma calcium levels were elevated because of pseudohypercalcemia (10). These case reports highlight the importance of considering pseudohypercalcemia in the differential diagnosis of hypercalcemia in multiple myeloma. Recognizing this condition is essential for avoiding misdiagnosis and ensuring appropriate management, and the measurement of ionized calcium should be integrated into clinical practice to enhance diagnostic accuracy and optimize treatment outcomes.

In conclusion, we report a rare case of a patient with pseudohypercalcemia associated with multiple myeloma. Although hypercalcemia is a hallmark feature of symptomatic myeloma, pseudohypercalcemia may arise because of calcium binding to abnormal immunoglobulins, as observed in this patient. In instances where elevated serum calcium levels are observed in the absence of clinical symptoms, pseudohypercalcemia should be considered, and the measurement of serum ionized calcium is essential for establishing an accurate diagnosis.
